# Surface treatments of the zirconia-reinforced lithium disilicate ceramic in the adhesion to the resin cement

**DOI:** 10.1590/0103-6440202405674

**Published:** 2024-03-22

**Authors:** Mirko A.R. Aguilera, Américo C. Bortolazzo, Lourenço Correr-Sobrinho, Rafael L. X. Consani

**Affiliations:** 1 Department of Restorative Dentistry, Division of Dental Material, Piracicaba Dental School, UNICAMP, SP, Brazil.; 2 Department of Prosthodontics and Periodontology, Division of Complete Denture, Piracicaba Dental School, UNICAMP, SP, Brazil.

**Keywords:** Ceramic, surface treatment, bond strength, surface characterization, failure, contact angle.

## Abstract

This study verified the effect of surface treatments of the zirconia-reinforced lithium disilicate ceramic bonded to resin cement. Ceramic blocks were divided according to treatments (n=10): FA+SRX (Fluoric acid + silane RX), FA+MDP (Fluoric acid + MDP), FA+SCF+MDP (Fluoric acid + silane CF + MDP), FA+MEP (Fluoric acid + MEP), and MEP (Self-etch primer). Resin cement cylinders were made in the ceramic blocks, photoactivated with 1,200 mW/cm² for 40s, stored in water at 37°C for 24h, and evaluated by the microshear strength test, optical failure descriptive analysis (%), surface characterization (SEM) and contact angle (Goniometer). Other samples were submitted to 10,000 thermocycles between 5°C and 55°C. Bond strength data were submitted to two-way ANOVA and Tukey’s test. Contact angle to one-way ANOVA and Games-Howell's test (5%). At 24h, MEP showed higher bond strength, and FA+SRX the lower. FA+MDP and FA+SCF+MDP showed similar values and FA+MEP was intermediate. After thermocycling, FA+SCF+MDP, FA+MEP, and MEP showed higher values, and FA+SRX the lower while FA+MDP was intermediate. When the periods were compared, FA+MDP, FA+SCF+MDP, FA+MEP, and MEP showed higher values for 24h while FA+SRX was similar. SEM showed retentive surface and crystal exposure when treated with FA+SCF+MDP. The less retentive surface was obtained with MEP, and the other treatments promoted intermediate irregularities. In conclusion, surface treatment and thermocycling promoted different values of adhesive strength and contact angle in a zirconia-reinforced lithium silicate ceramic. Failures were predominantly adhesive, and the ceramic surface was characterized by different levels of roughness and selective exposure of crystals.

## Resumo

O objetivo neste estudo foi verificar o efeito de tratamentos de superfície da cerâmica de dissilicato de lítio reforçada com zircônia fixada ao cimento resinoso. Os blocos cerâmicos foram separados de acordo com os tratamentos (n=10): AF+SRX (ácido fluorídrico + silano), AF+MDP (ácido fluorídrico + MDP), AF+SCF+MDP (ácido fluorídrico + silano + MDP), AF+MEP (Ácido fluorídrico + MEP) e MEP (Primer auto condicionante). Cilindros de cimento resinoso foram confeccionados em cada bloco cerâmico, foto ativados com 1.200 mW/cm² por 40 segundos, armazenados em água a 37°C por 24 horas e avaliados pelo teste de resistência ao micro cisalhamento, falha por análise descritiva em microscopia óptica (%), caracterização da superfície por imagens MEV e ângulo de contato (Goniômetro). Outras amostras foram submetidas a 10.000 ciclos térmicos (5° e 55°C). Os dados de resistência de união foram submetidos a ANOVA dois fatores e teste de Tukey (5%). Ângulo de contato com ANOVA um fator e teste de Games-Howell (5%). Em 24 horas, MEP apresentou maior resistência de união e AF+S a menor. AF+MDP e AF+SCF+MDP apresentaram valores similares e AF+MEP foi intermediário. Após a ciclagem térmica, AF+SCF+MDP, AF+MEP e MEP apresentaram valores maiores e AF+SRX o menor enquanto AF+MDP foi intermediário. Quando comparados os períodos, AF+MDP, AF+SCF+MDP, AF+MEP e MEP apresentaram valores maiores em 24 horas enquanto AF+SRX foi similar. MEV mostrou superfície mais retentiva e exposição de cristais quando tratada com AF+SCF+MDP. A superfície menos retentiva foi obtida com MEP, e os demais tratamentos promoveram irregularidades de superfície intermediárias. Conclui-se que o tratamento superficial e a termociclagem promoveram diferentes valores de resistência adesiva e ângulo de contato em cerâmica de silicato de lítio reforçada com zircônia. As falhas foram predominantemente adesivas, e a superfície cerâmica foi caracterizada com diferentes níveis de rugosidade e exposição seletiva de cristais.

## Introduction

The constant search for esthetic biocompatible materials with adequate mechanical properties remains an objective of restorative dentistry. Among these materials, dental ceramics stand out with chemical composition modified aiming to increase the mechanical strength, improve the adhesive condition, and provide adequate aesthetic characterization.

The appropriate association of these characteristics in relation to ceramics is not easily achieved in clinical practice. Vitreous or feldspathic-based ceramics with excellent aesthetic conditions do not show adequate and desirable mechanical strength to make indirect restorations in posterior teeth, limiting the indication of the material for some clinic prosthetic works.

Zirconia-based ceramics show satisfactory mechanical properties, reaching greater flexural strength values. However, they do not adequately reach the same optical properties levels of mineralized structures of the natural tooth. In addition, cyclic loading fatigue significantly reduces the force necessary to fracture the composite resin and all-ceramic crowns, whereas adhesive cementation significantly increases the fracture load value ^1^.

The development of glass ceramics infiltrated by alumina or alumina associated with magnesium or zircônia has shown satisfactory mechanical properties without damage to aesthetics for implant-supported zirconia crowns with sintered veneer, demonstrating good clinical performance ^2^. According to the ceramic manufacturer, the improvement in these properties was due to the development of lithium silicate ceramics infiltrated with 10% by weight of zirconia oxide crystals to prevent or decrease the formation and propagation of microcracks ^3^. This chemical formulation changed the percentage of the ceramic vitreous phase, facilitating the compatibility of the surface etching with fluoric acid, more effective than sandblasting with aluminum oxide particles and CoJet ^4^.

Different adhesive treatments have been proposed to bond dental ceramics to natural teeth, such as the etching of tooth mineralized surfaces and ceramics or different associations between adhesive systems and resin cement. In addition, the combination of chemical and mechanical treatments has been capable of improving the hydrolytic stability of the resin-ceramic bond which needs to achieve a clinic-satisfactory level ^5^.

Application of the silane to the glass ceramic surface after fluoric acid etching appears to be a suitable method for achieving satisfactory bonding between the composite resin and ceramics ^6^. The mechanical abrasion by aluminum oxide particles followed by the application of primer showed the highest bond strength level to enamel; therefore, the method can also be recommended as a promising surface treatment to achieve a durable bond with densely sintered zirconia ceramic ^7^. In addition, it was claimed that a resin-ceramic tensile resistance can be obtained by appropriate silane application without previous etching of the ceramic surface with fluoric acid ^8^.

On the other hand, several treatments have been proposed to increase the chemical activation of the surface through phosphate monomers, promoting a stable hydrolytic chemical bond with zircônia, forming a longer carbonyl chain, and causing a beneficial effect to increase the strength of the adhesive bond ^9^.

Due to the hybrid composition, the lithium silicate ceramics infiltrated with zirconia oxide would also be available for other etching protocols, promoting better results than those obtained with conventional methods ^10^. This procedure would depend on the action of zirconia oxide reacting with the phosphate monomer chemically composing the primer. Although the protocol is promising, the time required to perform the clinical procedure is longer, and the technique is considered complex and susceptible to the dentist's performance.

This fact could justify the association or development of new protocols or even the simplification of conventional treatments. As claimed by the manufacturer, the fluoric acid etching associated with the silane in a single step would promote greater strength of the bond between ceramics and resin cement. However, there are still no studies that confirm the effectiveness of the method in a longer time. Thus, establishing other suitable cementation protocols for modified glass ceramics aiming to increase the bond strength of indirect dental restorations would be desirable and timely.

This study aimed to verify the effect of surface treatments of the zirconia-reinforced lithium disilicate ceramics in the adhesion to resin cement, bond failure, surface characterization, and contact angle. The study hypothesis was that the different surface treatments would not influence the factors: 1) Adhesive strength, 2) Bond failure, 3) Surface characterization, and 4) Contact angle.

## Materials and methods

### Materials

The following materials were used in the study: Vita Suprinity zirconia-reinforced lithium silicate dental ceramic (Wilcos, Petropolis, RJ, Brazil) for CAD/CAM; FA - Fluoric acid gel (Maquira Dental Products, Maringa, PR, Brazil); SRX - Silane (RelyX Ceramic Primer [methacryloxypropyltrimethoxysilane], 3M ESPE, Sumare, SP, Brazil); MDP - 10-methacryloyloxydecyl dihydrogen phosphate monomer (Kuraray Noritake Dental, Sao Paulo, SP, Brazil); SCF - Silane (Clearfil Ceramic Primer Plus [3-trimethoxysilylpropyl methacrylate], Kuraray Noritake); MN (Phosphate ester monomer Monobond N), Ivoclar Vivadent, Barueri, SP, Brazil; MEP (Self-etching ceramic primer Monobond Etch & Prime [2-methacryloxyethyl dihydrogen phosphate], Ivoclar Vivadent).

The groups established were FA+SRX (Fluoric acid + silane RelyX Ceramic Primer; FA+SCF+MDP (Fluoric acid + silane Clearfil Ceramic Primer Plus + MDP; FA+MN (Fluoric acid + Phosphate ester monomer Monobond N); FA+MEP (Fluoric acid + MEP self-etching ceramic primer Monobond Etch & Prime, and MEP.

The protocols for surface treatment of the experimental groups were:

FA+SRX (Traditional fluoric acid application for 20s. Washing with water for 10s, air jet drying for 10s, and ultrasonic cleaning for 10 min. Silane application for 1 min and air jet drying for 10s).

FA+SCF+MDP (Traditional fluoric acid application for 20s. Washing with water for 10s, air jet drying for 10s, and ultrasonic cleaning for 10 min. MDP + silane application for 1 min and air jet drying for 10s).

FA+MN (Traditional fluoric acid application for 20s. Washing with water for 10s, air jet drying for 10s, and ultrasonic cleaning for 10 min. MN + silane application for 1 min, and air jet drying for 10s).

FA+MEP (Traditional fluoric acid application for 20s. Washing with water for 10s, air jet drying for 10s, and ultrasonically cleaning for 10 min. MEP actively rubbed for 20s, waiting for 40s, washing with water for 10s, and air jet drying for 10s).

MEP (MEP actively rubbed for 20s, waiting for 40s, washing with water for 10s, and ultrasonic cleaned for 10 min).

### Sample preparation

Zirconia-reinforced Vita Suprinity lithium silicate ceramic CAD/CAM material was cut with the diamond disk in a cutter (IsoMet Low Speed 1000; Buehler; Lake Bluff, IL, USA) obtaining blocks (6x7x1.5 mm) that were heated in oven (Austromat M; Dekema, Freilassing, Germany) under vacuum at 840°C and cooled slowly. The ceramic blocks of each group (n=20) were individually embedded in chemically activated acrylic resin (Classico; Sao Paulo, SP, Brazil) in rigid PVC tubes. The block bond surface was abraded with #600 silicon carbide sandpaper (Norton, Guarulhos, SP, Brazil) and cleaned with water in an ultrasonic vat (Dental Cremer; Blumenau, SC, Brazil) for 10 min. 

The adhesive (Single Bond Universal; 3M ESPE, Sumare, SP, Brazil) was actively applied to the ceramic bond surface with a microbrush for 20s followed by an air jet for 5s according to the manufacturer's recommendations. In each block, 4 cylinders (1.2 mm in diameter x 2 mm in height) were made with resin cement (RelyX Ultimate, 3M ESPE) using a silicone matrix (Express, 3M ESPE). An acetate strip was placed on the matrix filled with resin cement and submitted to a static load of 50g for 1 min. The resin cement cylinders were light-cured (Bluephase; Ivoclar Vivadent, Barueri, SP, Brazil) with an intensity of 1,200 mW/cm² for the 40s.

Samples of each group (n=10) were stored in deionized water in a microbiological oven (7Lab; Rio de Janeiro, RJ, Brazil) at 37°C for 24h. Other samples (n=10) were subjected to 10,000 thermal cycles (OMC250LC; Odeme, Luzerna, SC, Brazil) between 5°C and 55°C with permanence time in each water bath of 30s and transfer time of 30s.

### Microshear bond strength (µSBS)

A mechanical microshear bond strength test was performed in a universal testing machine (Instron; model 4411, Canton, MA, USA) with a 500 kgf load cell. The resin cement cylinder aligned perpendicular to the load cell during the test was pulled with a 0.2 mm diameter stainless steel wire (Morelli, Sorocaba, SP, Brazil) placed in the interface between the resin cement and ceramic. The tensile force was applied at a speed of 1 mm/min until failure.

### Bond failure

Bond failures were observed under an optical microscope (Carl Zeiss 475003-9902; Oberkochen, Germany) at 32x magnification and classified as adhesive, cohesive in ceramic, cohesive in cement, or mixed. A representative sample from each group was gold-plated and analyzed by SEM (JEOL JSM 5600 PV, Tokyo, Japan) at 50x magnification.

### Surface characterization

Morphological characterization of the ceramic surface was made in representative samples from each group. The sample surface was standardized with #600 silicon carbide sandpaper (Norton) and submitted to treatments: Fluoric acid + silane (FA+SRX); Fluoric acid+silane+MDP (FA+SCF+MDP; Fluoric acid + Phosphate ester primer (FA+MN); Fluoric acid + Self-etching ceramic primer (FA+MEP); and Self-etching ceramic primer (MEP). The samples were coated with gold and analyzed by SEM (JEOL JSM 5600 PV, Tokyo, Japan) at 4,000x magnification.

### Contact angle

Samples were analyzed in relation to the contact angle according to the following treatments: Untreated surface (SCS); Fluoric acid (FA); Fluoric acid+silane (FA+SRX); Fluoric acid+silane+MDP (FA+SCF+MDP); Fluoric acid + Phosphate ester primer (FA+MN); Fluoric acid + Self-etching ceramic primer (FA+MEP); and Self-etching ceramic primer (MEP).

After the ceramic treatment, a deionized water drop (3µL) was deposited on the sample surface with a syringe (n=16). After the 30s, the software coupled to the goniometer (Digidrop Contact Angle Meter; GBX, Bourg de Peage, France) provided the values of the right and left angles of the water drop on the sample surface, obtaining the arithmetic mean.

### Statistical analysis

Statistical analysis was performed with software (SigmaPlot 12.0, Systat software) and the assumptions of normal distribution and equality of variances were verified. The microshear strength values were analyzed by two-way ANOVA followed by Tukey's post hoc test for multiple comparisons (5%). Contact angle data were analyzed by one-way ANOVA followed by the Games-Howell post hoc test (5%). The bond failure and the surface characterization of the ceramics were evaluated using descriptive analysis of the images.

## Results

### Microshear bond strength (µSBS)

Table 1 shows the mean value and standard deviation of µSBS in relation to the surface treatments. Two-way ANOVA showed that treatment (p<0.001), thermocycling (p<0.001), and the association between them (p=0.007) influenced the µSBS values.

**Table 1:** Mean values of microshear strength and standard deviation (SD) about 24h and thermocycling treatments.

Treatment	µSBS (MPa)					
	24h (SD)			Thermocycling (SD)		
FA+SRX	24.41	(3.76)	Ca	21.76	(3.66)	Ba
FA+SCF+MDP	31.74	(5.89)	Ba	24.92	(3.13)	ABb
FA+MN	32.41	(4.91)	Ba	27.12	(1.88)	Ab
FA+MEP	36.09	(4.87)	ABa	28.34	(2.98)	Ab
MEP	40.70	(3.21)	Aa	29.01	(2.12)	Ab

Means followed by equal letters (capital in each column and lowercase in each row) do not differ by Tukey's test at 5%.

At 24 h, MEP showed higher bond strength (40.70 ± 3.21) and FA+SRX lower (24.41 ± 3.76). FA+SCF+MDP (31.74 ± 5.89) and FA+MN (32.41 ± 4.91) showed similar values, and FA+MEP (36.09 ± 4.87) was intermediate. After thermocycling, MEP (29.01±2.12), FA+MEP (28.34±2.98), and FA+MN (27.12±1.88) showed higher values, FA+SRX (21.76±3.66) lower, and FA+SCF+MDP (24.92±3.13) was intermediate. When the periods were compared, FA+SCF+MDP, FA+MN, FA+MEP, and MEP showed higher values at 24h compared to thermocycling. FA+SRX was similar in both times.

### Bond failure

Failure analysis is shown in Table 2. Higher values of mixed failures at 24h were observed for FA+SRX and FA+SCF+MDP, and the lowest for FA+MDP, FA+MEP, and MEP). The highest value of adhesive failure was observed for FA+SCF+MDP and the lowest for FA+MEP. The highest values of cohesive failures in ceramic occurred for FA+MEP. Cohesive failures in ceramic were not shown for FA+MDP and FA+SCF+MDP and few cohesive failures in cement occurred for FA+SRX, FA+MEP, and MEP.

After thermocycling, higher values of mixed failures were observed for FA+SRX and lower for MEP. The highest value of adhesive failures was observed for FA+MDP and the lowest for FA+MEP. The highest value of cohesive failure in ceramic occurred for FA+MEP. Few cohesive failures in cement were shown for FA+SRX and FA+MEP.

**Figure 1:**
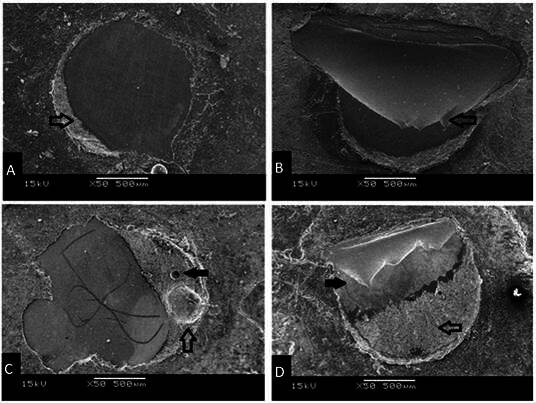
Representative micrographs of the failures: A - Adhesive, arrow indicates cement residue; B - Cohesive in ceramic, arrow indicates fracture outset; C - Cohesive in resin cement, white arrow indicates resin cement and black arrow shows pore in cement; and D - Mixed, white arrow indicates resin cement residue and black arrow shows ceramic and fracture.

Failure modes obtained in SEM images (A- Adhesive, B- Cohesive in ceramic, C- Cohesive in resin cement, and D- Mixed) are shown in Figure 1.

### Surface characterization

**Figure 2:**
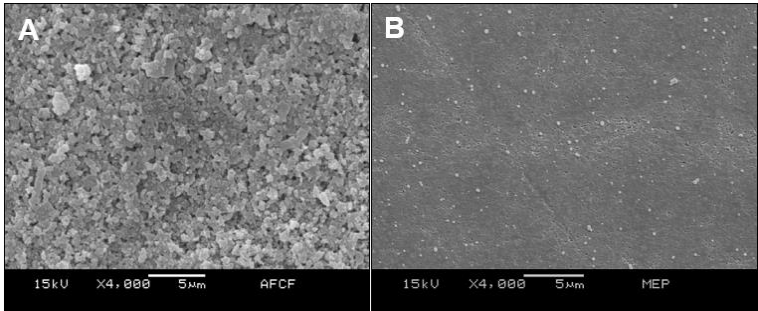
Ceramic micrographs for surface treatment: A - FA+SCF+MDP (4,000x) and B - MEP (4,000x).

Analysis of the micrographs obtained by SEM showed a surface with a more retentive pattern and selective exposure of crystals when treated with FA+SCF+MDP (Figure 2A). The less retentive surface was obtained with the MEP treatment (Figure 2B), and the other treatments promoted intermediate surface irregularities.

**Table 2:** Absolute number and percentage of failures.

Treatment		Failure absolute number and (%)			
		M*	A^#^	CCer^π^	CCem^ø^
24 h	FA+SRX	15 (38.5)	19 (48.7)	5 (12.8)	0 (0)
	FA+SCF+MDP	8 (20)	31 (77.5)	0 (0)	1 (2.5)
	FA+MN	14 (36.8)	23 (60.5)	0 (0)	1 (2.5)
	FA+MEP	8 (20.5)	2 (5.1)	29 (74.4)	0 (0)
	MEP	8 (20.5)	23 (59)	8 (20.5)	0 (0)
Thermocycling	FA+SRX	19 (48.7)	14 (35.9)	5 (12.8)	1 (2.6)
	FA+SCF+MDP	4 (10.3)	32 (82.1)	3 (7.7)	0 (0)
	FA+MN	8 (20)	27 (67.5)	5 (12.5)	0 (0)
	FA+MEP	7 (17.5)	5 (12.5)	26 (65)	2 (5)
	MEP	1 (2.6)	29 (74.4)	9 (23.1)	0 (0)

M* (Mixed); A^#^ (Adhesive); CCer^π^ (Cohesive in ceramic); CCem^ø^ (Cohesive in cement).

### Contact angle

A significant difference (p<0.001) for the contact angle values promoted by the etching treatments was observed. Table 3 shows the lowest value for FA+SRX and higher for FA+MEP with differences in relation to the other etching protocols. These other etchings showed intermediate values with a significant difference when compared between them.

**Table 3:** Mean values (standard deviation) of the contact angle for the surface treatment.

Treatment	Contact angle (º)	
SCS	35.9 (8.05)	d
FA+SRX	25.2 (8.78)	e
FA+SCF+MDP	36.7 (13.4)	de
FA+MN	54.9 (14.7)	c
FA+MEP	100.1 (16.9)	a
MEP	83.8 (5.42)	b

Means followed by distinct lowercase letters differ by the Games-Howell test (5%).

## Discussion

The results of Table 1 show that the adhesive strength values (MPa) were statistically different and influenced by the surface treatments (p<0.001) and thermocycling (p<0.001). Therefore, the study hypothesis that the adhesive bond strength would not be influenced by surface treatments and thermocycling was not confirmed. 

Different bond values promoted by the surface treatments occurred due to different primer abilities to form chemical bonds between silane and resin cement. In addition, the bond also depends on the different levels of mechanical interlocking of the resin cement into the surface ceramic irregularities. However, a previous study claims that the physicochemical adhesion would be considered stable in the long term since thermocycling and storage in water did not decrease the adhesion value when the silane was applied in feldspathic ceramic ^11^.

Whatever the retentive conditions of the ceramic surface, the chemical reaction between ceramic and silane was considered responsible for the higher shear bond strength ^12^. On the other hand, in the polished ceramic significantly lower bond strength and exclusively adhesive failure were observed ^10^. However, another interesting fact shown in a previous article was that different ceramic surface conditions did not negatively affect the bond strength due to the chemical conditioner's ability to form siloxane bonds when associated with acidic solutions ^13^.

Associating acid etching with a primer containing 10-MDP (10-methacryloyloxydecyl dihydrogen phosphate), FA+SCF+MDP and FA+MN showed higher values when compared to FA+SRX at 24h and after thermocycling. A previous study has shown that the phosphate ester monomer would be a promising adhesive method for zirconia ceramic ^14^. On the other hand, the ceramic types and the thermocycling did not affect, but the etching method had a significant effect on the shear bond strength when the higher value for fluoric acid compared to other surface treatments ^15^.

FA+MEP and MEP treatments provided higher values of bond strength at 24h (36.09 - 40.7, respectively) and after thermocycling (28.34 - 29.01, respectively). Fluoric acid associated with MEP resulted in higher tensile bond strength and surface roughness increase when compared to MEP applied alone, showing also higher number of adhesive failures between resin cement and ceramic. It is claimed that the bonding strength value is significantly impaired by the storage in water, and the etching with hydrofluoric acid resulted in a higher value of tensile strength and increased surface roughness compared to MEP treatment ^16^. This fact seems to indicate that the tensile value would be different between the types of ceramics, whatever the method of surface treatment.

The previous study showed that the self-etching primer promoted the highest bond strength and surface wettability decrease for feldspathic and lithium silicate ceramics. In addition, SEM and EDS analyses showed ceramics with similar components but different surface topographies, and the self-etching primer was able to promote a higher adhesive resistance compared to the fluoric acid associated with silane ^17^. These data collaborate with the current study results showing higher value for MEP at 24h. In contrast, there were values with statistical similarity when the samples of all treatments were submitted to thermocycling, except for FA+SRX.

The complexity of the effect of treatments on polished Vita Suprinity ceramics was demonstrated in a previous article when sandblasting and acid etching increased the shear strength compared to other ceramic types. Furthermore, the shear strength of samples subjected to Swiss and ISO tests was strongly correlated only for samples with adhesive failures ^10^. Statistically significant differences in roughness and surface energy were shown for all CAD/CAM ceramic materials with different surface treatments and SEM analysis showed structural damage considered to be material-dependent ^18^. These facts indicate that different chemical interactions can occur between primer and ceramic. Additionally, it would be possible to assume that the ceramic surface energy would also be different, promoting different adhesion values with the resin cement, as probably occurred at 24h with the MEP treatment.

Table 2 shows the highest values (%) of mixed failures at 24h and after thermocycling. Thus, the study hypothesis that the bond failure would not be influenced by the surface treatments and thermocycling was not confirmed.

The increase of cohesive failures in ceramic can be due to surface degradation caused by the acid etching weakening the mechanical interlocking strength of resin cement to ceramic. The treatment with acid etching is a dynamic process and its effect is dependent on the ceramic chemical constitution and surface topography. As a consequence, the acid etching promoted a decrease in the flexural strength of the low-melting feldspathic ceramic. Moreover, there is evidence that the surface alteration level would also be a function of etching time and acid concentration ^19^.

Bond strength decrease would be related to ceramic cohesive failures increase that occurred in 24h and after thermocycling for FA+MEP. The result contrasts with the cohesive failure amount in ceramic showed for MEP at 24h and after thermocycling. In addition, MEP also shows a higher value of adhesive failures at 24h and after termocycling when compared to FA+MEP.

It is claimed in previous work that the tension would be more strongly related to adhesive resistance than to the cohesive force of the materials, and the tensile and shear bond strengths are directly related to the geometry of the arrangement test and involved materials. In addition, the measurement force does not establish the mechanical properties of the materials, since its value is dependent on local conditions and the stress has little relationship with the force obtained. Thus, heterogeneous stresses are mechanically induced in the bond interface and focused in the resin cement, facilitating the occurrence of adhesive failures. The changes in bond strength value are also commonly related to different adhesive procedures.

Micrograph analysis obtained by SEM (Figure 2) shows that the microstructure of the ceramic surface resulting from FA+SCF+MDP etching is a relief with greater irregularities and retentive pattern with selective exposure of ceramic crystals that can be considered favorable to physicochemical bonding. However, that pattern was not evident for MEP, where there was probably less chemical interaction caused by the difficulty of the acid in dissolving the glassy phase of the ceramic, resulting in a surface with a more homogeneous appearance than that obtained for the material subjected to abrasion with silicon carbide sandpaper (SCS). On the other hand, although this study cannot confirm the presence of residues for methodological reasons, it is possible to infer that chemical residues are also present in treatments with acid etching.

The result seems to confirm the importance of the chemical bond caused by the primer. When the primer was applied after acid etching, a less retentive surface was observed, probably due to the residues filling the surface irregularities. Therefore, the study hypothesis that the surface characterization would not be influenced by the surface treatments and thermocycling was rejected.

Another interesting fact is there are divergencies and qualitative differences in the surface texture of ceramics submitted to etching treatments. As a consequence, excessive etching revealed more crystals and caused surface pitting for the longest period. However, the reconditioning changed the surface condition but did not completely remove the texture created by the initial etching ^20^. However, the silane-only containing primer and MDP and silane-containing primer can obtain better immediate and long-term shear bond strength for properly and excessively etched ceramic-coated zirconia, respectively ^21^.

Moreover, surface etching with fluoric acid, sandblasting with 50μm Al_2_O_3_ particles, sandblasting + Er: YAG laser irradiation, Er: YAG laser irradiation or tribochemical silica coating (CoJet Sand) followed by silane application significantly affected the surface texture of CAD-CAM ceramic. Surface treatment with CoJet revealed significantly higher bond strength compared to sandblasting in Y-TZP and monolithic zirconia ^4^.

Previous study shows that the adhesive failure occurred mainly for control and fluoridic acid etching groups, while silane, fluoridic acid etching + silane, and MEP demonstrated a greater amount of mixed failure. The micrographs showed a ceramic roughened surface promoted by the etching with fluoric acid, but no significant difference was found between the other surface treatments. On the other hand, the absence of surface treatment drastically reduces the µSBS between the ceramic and the characterization layer. Conditioning with 5% fluoric acid for 60s is the most suitable treatment for hybrid ceramic adhesion ^22^.

The contact angle promoted by the surface treatments is shown in Table 2. With a statistically significant difference in relation to the other groups, FA+MEP showed a higher value and the lowest value was for FA+SRX. The study hypothesis that the contact angle would not be influenced by the surface treatments was not confirmed.

Considering that the contact angle is a quantitative measure of the wetting of a solid by a liquid, in any situation the value of the contact angle to be obtained depends mainly on the relationship that exists between the adhesive forces of the solid and the cohesive forces of the liquid. The results obtained in the current study seem to confirm this assertion. With a statistically significant difference in relation to the other groups, FA+MEP showed a higher value and FA+S lowest value. The other treatments showed values with significant differences when compared to each other, while FA+SCF+MDP was intermediate between SCS and FA+SRX. The study hypothesis that the contact angle would not be influenced by the surface treatments was not confirmed.

Previous studies showed that the contact angle would be influenced by the surface conditions since a significant increase in roughness and wettability was observed with the increase in the fluoric acid etching time. It is also important to note that after the acid etching, there was an increase in the area available for bonding and the surface energy, improving the interfacial tension, the chemical interaction with the silane, and the adhesion level. Chemical etching promoted significant changes in the number and poros pattern, crystalline structure, surface roughness and wettability with etching time increased. Etching for a short time resulted in small pores, and a longer time promoted wider and irregular grooves. A significant increase in the surface roughness and wettability was shown with the etching duration increase, suggesting a strong correlation between roughness and wettability ^23^. On the other hand, the bond failures in different self-etching systems were predominantly adhesive ^24^, and the contact angles were similar ^25^. These results appear to be constant in adhesive bonding procedures.

However, it is still necessary to assess carefully each treatment for adhesive bonds using methods that attempt to characterize the interface energy. The adhesive bonding associates physical, chemical, and mechanical contributions, and also it is strongly based on micro-mechanical interlocking. Interface characterization before adhesion, during clinical service, and after failure yet remains a challenge for the researchers ^26^. The type and composition of the specific ceramic determine the selection of the most effective bonding protocol, including surface pretreatment followed by application of primer. Understanding ceramic properties and chemical compositions enables the clinician to make proper material selection decisions for clinically successful and long-lasting restorations ^27^.

A study claims that submitting the ceramic to 10,000 thermal cycles would promote a lower value of adhesive strength in most of the protocols evaluated ^15^ ^,^ ^28^ ^,^ ^29^ due to the hydrolytic degradation of the polymers composing the resin cement, causing oxidation, degradation of functional groups, and cleavage of the material structural chain ^30^.

Although the results obtained in 24h provide evidence that allows us to understand the effect of surface treatments and thermocycling on the adhesion, bond failure, surface characterization, and contact angle of a zirconia-reinforced lithium silicate ceramic, the study limitation did not permit the correlation among these factors in longer time of use, as well as to verify the effect on maintenance or degradation of the adhesive bond.

## Conclusion

Regardless of the study's limitations, the following conclusions can be considered:

1- Surface treatment and thermocycling promoted different values of adhesive strength and contact angle in a zirconia-reinforced lithium silicate ceramic.

2- Failures were predominantly adhesive, and the ceramic surface was characterized by different levels of roughness and selective exposure of crystals.
